# From first generation to the next: evolution and research trends in iStent technology

**DOI:** 10.3389/fmed.2025.1724886

**Published:** 2026-01-23

**Authors:** Bryan Chin Hou Ang, Natalie Shi Qi Wong, Bjorn Betzler, Sheng Yang Lim

**Affiliations:** 1 Department of Ophthalmology, Tan Tock Seng Hospital, National Healthcare Group Eye Institute, Singapore, Singapore; 2 Lee Kong Chian School of Medicine, Nanyang Technological University, Singapore, Singapore; 3 Department of Ophthalmology, Mayo Clinic, Jacksonville, FL, United States

**Keywords:** glaucoma - surgery, iStent, iStent inject, iStent inject W, minimally invasive glaucoma surgery, primary open angle glaucoma

## Abstract

The iStent series constitutes a range of trabecular bypass minimally invasive glaucoma surgery (MIGS) devices, which offers intraocular pressure (IOP) reduction with favourable safety profiles, in patients with open-angle glaucoma (OAG). Having undergone significant evolution since its initial US FDA-approval in 2012, successive generations address previous limitations, while enhancing IOP-lowering efficacy through device and delivery system design iterations. Longer-term and real-world iStent data demonstrate the durability of IOP- and medication-lowering outcomes with minimal complications, while preliminary studies across a wider spectrum of glaucoma subtypes and severities provide limited evidence of successful outcomes beyond mild-to-moderate OAG, both with and without concomitant cataract surgery. Aqueous humour outflow assessment and novel intra-operative techniques may further facilitate more accurate and effective iStent positioning. Despite typically higher upfront costs, results from both cost-effectiveness and patient-reported outcome studies are encouraging. Combination MIGS with the iStent, leveraging on the multiple mechanisms of actions of various procedures, may provide greater IOP-lowering efficacy without compromising safety. With expanding clinical data and progressive enhancements, iStent technology is likely to remain a key component of the evolving MIGS landscape.

## 1 Introduction

Glaucoma, a progressive optic neuropathy with characteristic structural and functional visual field loss, is the most common cause of irreversible blindness worldwide (1, 2). Intraocular pressure (IOP), the only known modifiable risk factor to prevent disease progression, remains the target of glaucoma therapy (3).

Minimally invasive glaucoma surgery (MIGS) is gaining rapid traction globally, characterized by impressive safety profiles with minimal anatomical disruption and rapid recovery compared to traditional filtering surgeries (4). MIGS can be categorized into angle-based, suprachoroidal, and subconjunctival MIGS, based on their respective targeted sites of action (5).

Angle-based MIGS target the trabecular meshwork, identified as the site of greatest resistance to aqueous outflow in primary open angle glaucoma (POAG). They encompass trabecular-bypass stents [e.g., iStent series (Glaukos Corporation, Laguna Hills, CA, USA), Hydrus Microstent (Ivantis, Inc., Irvine, CA, USA) (6, 7)], trabecular excisional procedures [e.g., Kahook Dual Blade (New World Medical, Rancho Cucamonga, CA, USA), goniotomy, gonioscopy assisted transluminal trabeculotomy (GATT) (8, 9)] and canaloplasty procedures [e.g., iTrack microcatheter (Nova Eye Medical, Fremont, USA) (10)].

The iStent was the first MIGS implant to attain U.S. Food and Drug Administration (FDA) approval in 2012 (11). Today, the iStent series is used worldwide, and remains to date, the smallest FDA-approved implant in the human body (12). With an impressive safety profile, the iStent series has demonstrated successful reduction in IOP and medication burden while limiting disease progression in patients with mild-to-moderate OAG (13). Subsequent generations of the iStent (iStent inject, iStent inject W, iStent infinite) have been developed, with the stent design and implementation mechanism of each successive iteration addressing the limitations of its predecessor, while improving predictability of implantation and enhancing surgical efficacy.

This narrative review traces the evolution of iStent technology from its inception to the present day and examines five major themes of research and innovation over the past decade, summarized by Table 1.

**Table 1:** Summary of key research and innovation themes in iStent technology over the past decade.

Theme	Current evidence	Clinical implications	Areas of further research
Long-term outcomes	Short-term pivotal trials have established the safety and efficacy of iStent technology over 12–24 months. Subsequent mid-term studies and registry data report sustained IOP and medication reductions up to 5 years post-operatively, with low complication rates.	iStent technology provides safe and durable IOP and medication-lowering, both as a standalone procedure or in conjunction with cataract surgery. Data supports earlier interventional use in the disease course, to improve vision-related quality of life.	Longer-term outcomes beyond five years and across diverse populations and glaucoma types (e.g., NTG, advanced disease, eyes on maximal medical therapy, ethnicities with lower representation in pivotal trials) require further exploration.
Device design and technique	Studies demonstrate a dose–response effect with multiple iStents, supporting the transition from single- to multi-stent generations. Device design has evolved to reduce malposition and over-implantation risk – with wider flanges, improved delivery systems, and the option for multiple implantation attempts in newer models. Adjunctive imaging and intra-operative clinical signs have been proposed to aid confirmation of accurate placement. Both in- and ex-vivo studies suggest stent location may influence local aqueous humour outflow patterns and inform optimal stent location.	These advancements are likely to improve iStent implantation predictability and consistency, however, later design iterations require comparative and longer follow-up data.	The extent of additional clinical benefit in utilizing adjunct imaging and other clinical methods to ensure optimal stent position remains uncertain.
Expanded Indications and predictors	Early trials have previously supported the use of iStent technology in conjunction with cataract surgery in mild-to-moderate POAG. Subsequent studies demonstrate efficacy with standalone implantation and in advanced disease as well. Beyond POAG, iStent technology demonstrates modest IOP reduction in NTG, PACG, PXF and pigmentary glaucoma. However, higher complication rates were observed in PACG cohorts.	Limited, current evidence may form basis for consideration of iStent technology in a wider spectrum of glaucoma patients, particularly in Asian populations, where NTG and PACG are more prevalent. However, to date, these indications remain off-label. Growing recognition of possible predictive factors such as baseline IOP, prior interventions such as SLT, and preoperative medication load and duration, may help refine patient selection and set realistic outcomes expectations.	Further data is needed on longer-term outcomes in NTG, PACG, advanced disease and secondary glaucoma. Questions persist regarding identification of biomarkers for patient selection, and how preoperative factors may be systematically integrated into surgical selection.
Cost- effectiveness and patient-reported outcomes	Beyond benefits in IOP and medication reduction, studies demonstrate favourable cost-utility profiles for iStent technology, with long-term QALY gained despite higher upfront costs. Patient-reported measures demonstrate improvements in vision-related QOL and overall satisfaction, the result of a reduced treatment burden and lessened ocular-surface disease.	Clinicians may incorporate emerging evidence into patient counseling, highlighting the post-operative benefits of convenience, comfort, and reduced treatment burden, alongside traditional efficacy. Superior cost-effectiveness outcomes compared to alternative therapies may motivate the wider incorporation of MIGS into formal clinical practice guidelines and direct healthcare expenditure into appropriate subsidies to ensure greater accessibility.	Further health economics studies are needed to validate cost-effectiveness in lower-resource settings where upfront device cost may be a barrier, as well as in specific glaucoma types (NTG/PACG). Patient-reported outcome measures and metrics remain heterogenous, although there have been efforts to streamline MIGS QOL questionnaires.
Combination MIGS	Studies combining iStent technology with other MIGS procedures, such as endoscopic cyclophotocoagulation or canaloplasty, suggest additive IOP- and medication-lowering effects, compared with iStent alone. Comparative studies suggest that these combinations may achieve outcomes similar to bleb-forming MIGS in the short to medium term, with fewer serious complications.	Combination MIGS offers surgeons an expanded toolkit for patients requiring greater IOP reduction than a single procedure typically delivers, while still maintaining the safety advantage of a single, minimally invasive procedure. These combinations may support a paradigm shift toward more effective earlier surgical intervention, targeting multiple mechanisms of action.	Prospective data is lacking, particularly beyond two years post-operatively – hence, the durability of benefit of combination MIGS, relative to single devices or more invasive surgery, remains unclear. “Optimal” combinations of MIGS have yet to be determined. The incremental benefit of combining MIGS procedures appear to be modest, raising questions, and cost impact requires further evaluation.

### 1.1 Methods

A systematic literature search across electronic databases (PubMed, Embase, and Web of Science) was conducted to identify peer-reviewed articles on the iStent, iStent inject and iStent infinite. The search covered publications from inception to 28 July 2025. Keywords included: “iStent,” “iStent inject,” “iStent infinite,” “trabecular micro-bypass,” “minimally invasive glaucoma surgery,” and “MIGS.” Boolean operators and MeSH terms were used where appropriate to maximize sensitivity. Inclusion criteria were: (1) original studies (including pilot studies, cohort studies, observational studies, randomized controlled trials and case reports or series) focused on the iStent, iStent inject or iStent infinite that (2) reported clinical outcomes, safety, efficacy, or device comparisons, and were (3) full-text articles published in English. Exclusion criteria were: (1) non-English language publications, (2) conference abstracts, letters, editorials, and (3) animal studies and *in vitro* research.

## 2 iStent technology—A story of evolution

### 2.1 1st generation iStent

The 1^st^ generation iStent is a 1 mm single, heparin-coated, non-ferromagnetic titanium stent, designed with a self-trephining tipped snorkel and three retention arches, pre-loaded into a single-use injector. It works by incising and stenting the Schlemm’s canal (SC) to increase trabecular flow (14). It is offered concurrently with cataract surgery to patients with mild-to-moderate OAG, in the absence of angle abnormalities that may affect stent positioning (15). The pivotal trial published by Sameulson et al. in 2011 demonstrated that 66% of eyes with mild-to-moderate OAG which underwent combined iStent and cataract surgery achieved ≥20% IOP reduction at 1 year, compared to 48% of eyes which underwent cataract surgery alone, with similar rates of adverse events between both groups (15).

### 2.2 iStent inject and iStent inject W

**Figure 1:**
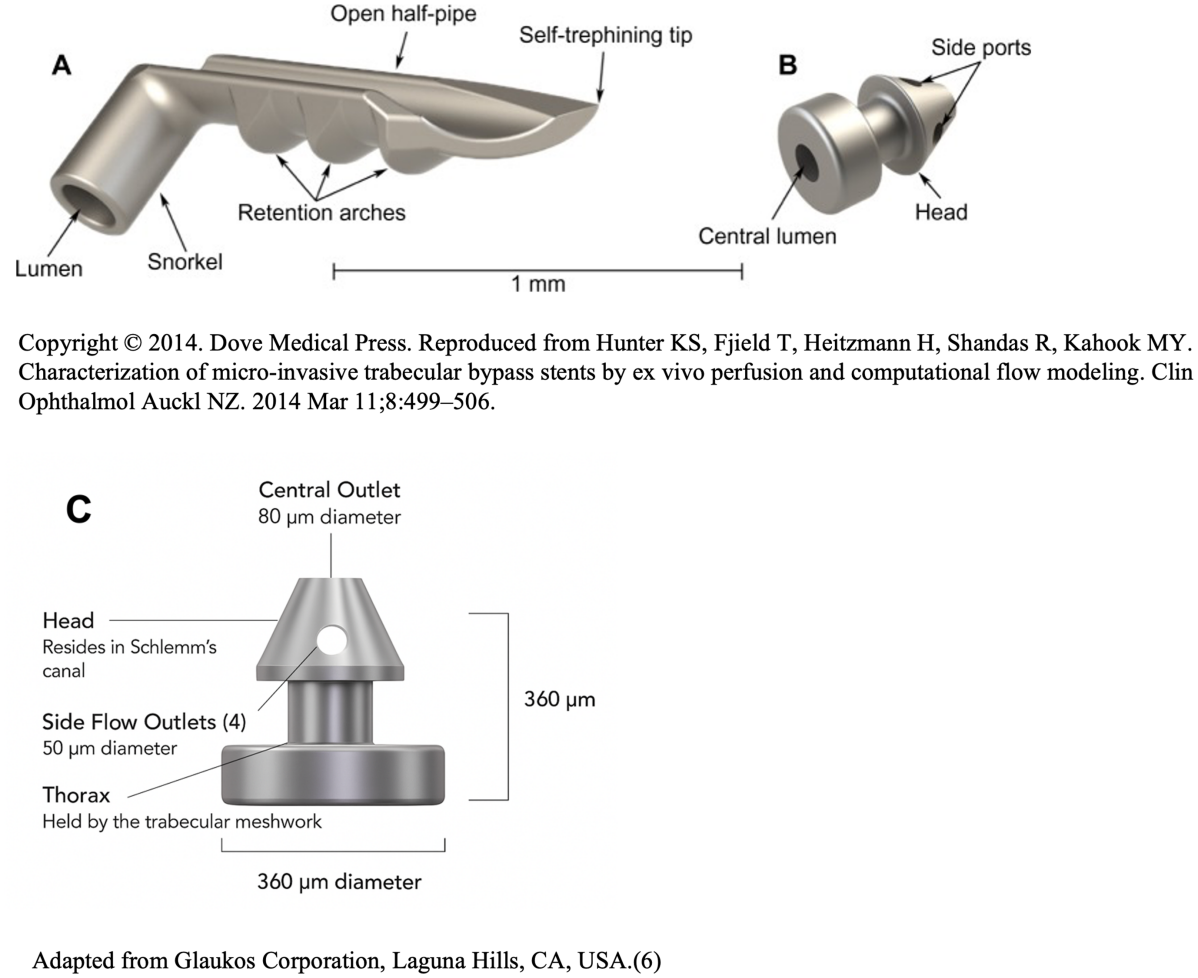
Illustrations of the **(A)** 1st generation iStent, **(B)** 2nd generation iStent inject, and **(C)** 3rd generation iStent inject W.

The 2^nd^ generation iStent inject, FDA-approved in 2018, features two shorter stents, measuring 360 μm in height and 230 μm in diameter, with a tapered head and a wide flange that sits in the anterior chamber. Both stents are placed 2–3 clock hours apart in the inferonasal quadrant of the angle, with their multidirectional design enabling them to deliver access to multiple collector channels and arcs of flow spanning up to 5–6 clock hours (6, 16). Indications for the iStent inject, offered in conjunction with cataract surgery to patients with mild-to-moderate OAG, were similar to its predecessor (17). The pivotal trial published by Sameulson et al. in 2019 found that after 2 years, 75.8% of eyes with mild-to-moderate OAG which underwent iStent inject combined with cataract surgery had ≥20% IOP reduction from baseline and 84% were drop-free; compared to 61.9 and 67%, respectively, in eyes which underwent standalone cataract surgery. Following studies replicated these outcomes, with an IOP reduction of 14.1–16% and a decrease of 0.61–1 medications reported 12–24 months after combined iStent inject and cataract surgery (18–20). The iStent inject has also been shown to result in greater IOP reduction compared to the 1^st^ generation iStent (21–23). Safety-wise, a meta-analysis of 1,159 eyes reported that for both the iStent and iStent inject, post-operative IOP spike was most common, reported in 12/27 (44.4%) studies; followed by stent obstruction and malpositioning, reported in 8/27 (29.6%) and 7/27 (25.9%) studies, respectively (24). The iStent inject W, introduced in 2020, has a design almost identical to the iStent inject, except for a wider, 360 μm flange. The iStent inject W was designed to increase IOP and medication reductions compared to the iStent inject, while providing improved device positioning consistency, without significant differences in postoperative complications (25). Figure 1 depicts illustrations of the 1st generation iStent, iStent Inject and iStent inject W.

### 2.3 iStent infinite

The iStent infinite, FDA-approved in 2022, is the latest iteration of the iStent series. The multi-use delivery system is pre-loaded with three iStent inject W microstents, designed to create arcs of flow spanning up to 8 clock hours. Sarkisian et al. demonstrated that in 76.1% of OAG eyes uncontrolled by prior therapy, standalone iStent infinite implantation achieved ≥20% IOP reduction from baseline without increasing anti-glaucoma medications, with a mean IOP reduction of 5.9 mmHg, after 1 year (26). Interim analysis of the ongoing 24-month INTEGRITY study reported that at 6 months, the iStent infinite had a greater proportion of eyes achieving ≥20% IOP reduction from baseline, compared to the Hydrus microstent (78.2% vs. 65.0%), with lower complication rates (3.3% vs. 16.9%). “Improper stent placement” was observed in lower frequency with the iStent infinite in 1/91 eyes, compared to 7/89 eyes with the Hydrus Microstent (1.1% vs. 7.9%) (27).

## 3 Key research and innovation themes in iStent technology over the past decade

### 3.1 Longer-term and real-life surgical outcomes

Acknowledging that the FDA pivotal trials on iStent technology were pharmaceutical-sponsored (15, 26, 28), these studies reported positive 12 to 24-month surgical efficacy outcomes, with good overall safety profiles. However, notably, Araújo et al. highlighted that compared to real-world untreated POAG patients, subjects in the pivotal iStent inject trial appeared to have higher unmedicated baseline IOPs, in both the treatment and control arms (29). The Early Manifest Glaucoma Trial, for example, found that on population-based screening, the mean baseline IOP was only 20.6 mmHg among patients with newly diagnosed OAG (30). The higher baseline IOPs in the iStent FDA pivotal trial may have resulted in higher IOP reductions after standalone phacoemulsification in the control arm, with only a mild additional 1.6 mmHg IOP reduction and 0.4 medication reduction after 24 months, favouring combined iStent inject implantation and phacoemulsification. Questions have also been raised on whether the IOP-lowering effects of phacoemulsification, possibly inflated by the high baseline IOPs amongst recruited patients, may have driven the reduction in IOP after combined iStent inject implantation and phacoemulsification; and whether surgical efficacy would be longstanding, given that 38% of controlled POAG eyes may lose IOP control 1 year after cataract surgery (29, 31).

Later studies attempted to replicate the outcomes of the pivotal trials and explore the durability of iStent technology in real-world settings. Longer-term studies on combined iStent device implantation and phacoemulsification, examining outcomes at 5 years or more, have reported sustained medicated IOP and medication reductions of 12.7–39% and 0.5–1.74, respectively (32–36). Complication rates in all iStent studies have remained low – the most common being transient IOP spikes or hyphema (32–36). The 5-year follow-up safety study of the pivotal iStent inject study found no device-related adverse events or complications, with the rate of endothelial cell loss remaining similar to controls (37). Data from large real-world registries also appears consistent with findings from these studies. 24-month data from the Intelligent Research in Sight (IRIS®) Registry of 1,435 eyes which underwent combined iStent inject and phacoemulsification showed a moderate 22% reduction in medicated IOP and a reduction of 0.86 medications, in eyes that had a baseline IOP of >18 mmHg (38). Despite the encouraging data emerging from studies following the pivotal trials, the retrospective, unmasked design of these real-world studies may limit the strength of any causal inferences, requiring appropriate caution when interpreting their results. Longer-term safety data may also be needed to clarify the theoretical longer-term risks of peri-implant fibrosis within Schlemm’s canal and its impact on subsequent filtration surgery.

### 3.2 Optimization of device design and implantation technique

While the 1^st^ generation iStent was delivered as a single device, studies have demonstrated a positive dose–response relationship between the number of stents and IOP-lowering efficacy. Belovay et al. attempted insertion of two or three 1st generation iStents combined with cataract surgery, and suggested multiple stents may result in greater IOP and medication reductions (39). Katz et al. (40) also reported that while the standalone implantation of one, two or three 1st generation iStents were all clinically effective, the implantation of more iStents was associated with incrementally greater IOP reductions and a reduced need to escalate medications after 42 months. These studies have supported the evolution of the single 1^st^ generation iStent to current multi-stent iterations.

Design changes of the iStent in successive iterations were likely driven by the intention to improve safety and implantation predictability. Multiple studies on the 1st generation iStent reported early post-operative stent occlusion and malpositioning in 2.6–18% of eyes, with 4.5–11.3% requiring secondary surgical intervention (11, 15, 39, 41–49). The implantation technique for the 1st generation iStent – insertion at an angle, and then sliding sideways (50) – creates torque, which may increase risk of malpositioning (51). The iStent inject abandoned the snorkel design, employing instead a unidirectional entry path into the SC without torsion, hence likely reducing the risk of stent malpositioning initially faced by its predecessor (51).

Over-implantation, however, continued to be observed with the iStent inject. Gillman et al. reported that up to 45.7% of iStent inject devices could be entirely burrowed within the angle, as detected on anterior segment optical coherence tomography (OCT), which was greater than that suggested by gonioscopy (52). Corrective intra-operative techniques to manage over-implantation were subsequently described, via the extraction and reimplantation of over-implanted stents with the use of either the delivery system (53) or microforceps (54). The iStent inject W was subsequently developed, featuring a wider 360 μm flange to reduce the risk of over-implantation and to improve stent visualization (6). Its accompanying delivery system was also modified to improve intra-operative visualization and more consistent iStent delivery. The latest iStent infinite delivery system now allows for unlimited stent delivery attempts, further facilitating intra-operative rectification of malpositioned stents.

There has been growing recognition that malpositioned iStents may result in suboptimal IOP-lowering outcomes. Gillman et al. found that malpositioned stents resulted in greater distances between the head of the stent and the SC, smaller SC dilatations, and ultimately higher post-operative IOPs (52). Novel intra-operative techniques have been described to enhance and confirm the accuracy of iStent placement. Ang et al. suggested two intra-operative clinical signs to confirm accurate placement of the head of the iStent in the SC—blood reflux from the iStent lumen, elicited by gentle anterior chamber decompression; and the “Schlemm’s bidirectional fluid wave,” observed within the canal segments adjacent to the iStent after flushing with balanced salt solution (55). Sarohia et al. (56) described visualization of aqueous flow in the external vasculature near the iStent as an independent predictor of long-term IOP or medication reduction. The use of adjunct imaging, including intra- and post-operative OCT, for real-time and early post-operative evaluation of iStent positioning, has also been described (52, 57–59).

Apart from optimising the positioning of the stent, other studies sought to determine the optimal location to achieve the greatest improvement in aqueous outflow after iStent insertion—by examining localized changes in aqueous outflow, before and after iStent implantation. Bostan et al. (60) suggested that episcleral venous outflow could be used to evaluate iStent patency, and Fellman et al. (61) examined downstream patterns of episcleral venous fluid waves in-vivo as a measure of aqueous outflow, concluding that they were observed 2 clock hours adjacent to implanted iStents, favouring the inferonasal region. Lusthaus et al. (62, 63) found sectoral improvement of nasal and temporal aqueous outflow via in-vivo hemoglobin video imaging, following iStent implantation. These studies suggest segmental improvement of aqueous outflow following iStent insertion and may support the current common practice of iStent insertion into the nasal region of SC (64). Furthermore, ex-vivo studies using sequential aqueous angiography showed that placing trabecular micro-bypass stents into regions that were initially angiographically silent led to increased angiographic outflow signals (65), suggesting its potential use in pre-operative planning. However, these imaging techniques to visualize aqueous flow may not yet be feasible for routine clinical practice. Furthermore, with the current trajectory of advancements in iStent technology, it is possible that targeted stent placement may be less relevant, as the stents evolve to recruit a greater breadth of collector channels to create larger arcs of flow.

### 3.3 Expansion in indications and study into predictors of surgical success

While FDA-pivotal trials supported its initial use in conjunction with cataract surgery in mild-to-moderate POAG, the iStent inject has also been applied in several off-label indications such as standalone surgery and even in advanced POAG. A meta-analysis of 778 eyes that underwent standalone iStent or iStent inject implantation reported a weighted mean IOP reduction of 30.9% and a mean reduction of 1.19 medications at 36–60 months (66). Single-arm studies have further shown that standalone iStent infinite surgery is effective in moderate-to-advanced OAG uncontrolled by prior surgical or medical therapy (26).

Aside from POAG, iStent technology has also been applied in normal-tension glaucoma (NTG), primary angle-closure glaucoma (PACG) and secondary OAG. Modest IOP reductions of 0.7–2.31 mmHg in NTG eyes with 1–1.2 medication reductions 12 months after combined iStent and phacoemulsification have been reported, commensurate with the lower baseline IOPs in NTG, compared to POAG eyes (67–69). In PACG eyes, significant IOP and medication reductions of 27% and 11%, respectively, have been reported after combined iStent and phacoemulsification compared to matched standalone phacoemulsification controls, albeit with higher rates of complications such as iris occlusion (27%) and hyphema (18.9%) compared to that previously described in OAG eyes (70, 71). Clement et al. further reported sustained IOP and medication reductions 3 years following combined iStent and phacoemulsification, across multiple types of mild-to-advanced glaucoma, including pseudoexfoliative and pigmentary glaucoma (72). These findings may be of particular relevance in Asian populations, with a greater prevalence of NTG and PACG (73–75). However, current evidence describing the iStent’s effectiveness and safety profile outside of mild-to-moderate POAG mostly stem from small, single-centre, frequently uncontrolled studies. To date, the use of iStent in these settings is still considered off-label, with preliminary data suggesting weaker efficacy and higher complication rates. Larger, adequately powered randomized controlled trials are therefore required to define the role of the iStent in NTG, POAG, and advanced POAG, and to characterize its risk–benefit profile in these off-label indications.

Researchers have also explored pre-operative factors which may affect the success of iStent implantation and also better inform patient selection. Tan et al. reported that prior SLT was associated with an increased risk of failure after combined iStent inject and phacoemulsification compared to matched controls. The risk of failure, however, was significantly reduced if patients had higher baseline IOPs (76). Multivariate analysis conducted by Morita et al. found that a high pre-operative IOP was associated with a lower likelihood of surgical failure for both the iStent and iStent inject W, while the surgeon appeared to also be a significant factor in the iStent inject W group (77). A higher medication burden (≥3 types) prior to standalone iStent implantation has also been shown to increase risk of surgical failure (78). Laboratory studies have demonstrated that prolonged use of anti-glaucoma medications may cause trabecular meshwork damage, which may blunt the IOP response after iStent implantation (79, 80).

### 3.4 Cost-effectiveness, patient-reported outcome measures and quality of life outcomes

Further to the traditional efficacy outcomes of IOP and medication reduction, other outcome measures, including health-economic metrics, patient-reported outcomes and quality-of-life measures, have also been studied. Cost-utility analyses have found that while combined iStent inject and phacoemulsification resulted in higher total lifetime costs compared to standalone phacoemulsification, combined surgery demonstrated significantly higher gains in terms of the number of blind eyes avoided and quality-adjusted life years (QALYs) gained (81, 82). iStent inject with or without phacoemulsification also provided greater QALY gains compared to standard clinical care (i.e. topical medications or laser therapy) and yielded lower cumulative total healthcare costs (83–86). Overall, these studies demonstrate that the greater upfront cost of iStent technology may be offset by better long-term QALYs and greater healthcare cost savings. Furthermore, better ocular surface disease index scores have been reported, attributed to reduced medication burden and exposure (87–89). There was improved vision-related quality of life, convenience, and patient satisfaction, demonstrated in improved visual function questionnaire scores (87, 90).

### 3.5 Comparative outcomes between the iStent and other MIGS

Head-to-head comparative data between the iStent series with other MIGS remain limited and confined to small, industry-sponsored cohorts. Meta-analyses of current limited data suggest that the iStent had a more favourable safety profile but slightly lower IOP-lowering efficacy compared to other Schlemm’s canal-based procedures such as the Hydrus Microstent, albeit with small effect sizes (91–93), and similar IOP-lowering efficacy with Kahook Dual Blade Goniotomy (94). Sufficiently-powered, independently-funded randomized controlled trials directly comparing iStent technology with other MIGS remains a key unmet need – the results of these studies would aid significantly in more precise patient selection for each MIGS device and procedure.

### 3.6 Combination MIGS with the iStent

Further advances in MIGS have led to the conceptualisation of combined MIGS (cMIGS). Many involve combining iStent technology with other MIGS, with the intention of targeting multiple complementary IOP-lowering pathways simultaneously.

One such combination is the iStent inject and endoscopic cyclophotocoagulation (ECP) procedure, also known as ICE2, in which the iStent and ECP are combined with phacoemulsification. The iStent inject enhances trabecular outflow by bypassing the trabecular meshwork, while ECP reduces aqueous humour production by ablating ciliary processes, producing synergistic IOP-lowering effects that have been reported in prospective studies (95). Pantalon et al. found that ICE2 achieved a greater IOP reduction than combined phacoemulsification and iStent inject (35% vs. 21%), with a lower final mean IOP (13.05 vs. 14.09 mmHg) and medications (1.24 vs. 1.39) after 12 months (96). Safety profiles were similar in both groups. ICE2 has also been compared to both minimally invasive bleb surgery (MIBS) and combined MIGS in terms of efficacy and safety. Qidwai et al. found that eyes undergoing ICE2, PreserFlo Microshunt (Santen Pharmaceutical Co., Ltd., Osaka, Japan) and XEN45 gel stent (Allergan Inc., CA, USA) implantation had no statistically significant differences in IOP or number of anti-glaucoma medications after 24 months of follow-up, with only transient post-operative complications in all groups (97). Helwe et al. showed that while ECP and Kahook Dual Blade trabeculectomy (PEcK) achieved greater mean IOP (5 vs. 3.14 mmHg) and medication reductions (1.35 vs. 1.01) than the ICE2 group, early post-operative hyphema was more frequent in the PEcK group (98). These findings suggest that while ab interno trabeculectomy creates a larger outflow window, the incremental benefit over iStent implantation may be modest and accompanied by greater bleeding risk. Hence, ICE2 may offer an alternative for eyes requiring greater IOP reduction, while avoiding risks and complications associated with more invasive techniques such as trabeculectomy.

The iStent series has also been combined with ab-interno canaloplasty, which lowers IOP by enhancing circumferential outflow via viscodilation and 360° catheterization of the SC, while the iStent, a trabecular meshwork bypass device, synergistically allows aqueous to enter the dilated SC more efficiently. In a retrospective analysis, Heersink and Dovich reported that canaloplasty with the 1^st^ generation iStent resulted in a larger mean IOP reduction at 6 months (2.9 ± 3.6 vs. 1.7 ± 3.1 mmHg), with more eyes achieving ≥20% IOP reduction and an IOP of <18 mmHg, compared to iStent implantation alone (46% vs. 35%), both with similar medication reductions (99). However, prospective data is still lacking for iStent combined with ab-interno canaloplasty.

Overall, early evidence suggests that cMIGS has potential to deliver incremental IOP-lowering efficacy compared to standalone iStent implantation. However, this additional benefit may be modest. Hence, patient selection is crucial and cMIGS should be reserved for eyes needing greater IOP reduction, justifying the additional procedural complexity and cost. At this time, current evidence remains limited and short-term, and the cost impact has not yet been well-evaluated.

## 4 Conclusion

In conclusion, iStent technology has undergone meaningful evolution since its introduction, with successive generations attempting to address previous limitations, while enhancing IOP-lowering efficacy through device and delivery system design iterations. Novel methods to facilitate and confirm optimal stent placement may improve surgical outcomes in addition to design iterations. Emerging longer-term, real-world data has demonstrated efficacy in a wider spectrum of glaucoma patients with varying subtypes and severities; however, these indications are considered off-label and current evidence remains limited. Data from imaging studies may better inform patient selection and facilitate targeted device implantation. Encouraging results from cost-effectiveness studies, incorporating patient-reported outcomes, may support the wider adoption of iStent technology. Finally, cMIGS involving iStent technology may have potential in leveraging on the favourable safety profile associated with MIGS, while employing multiple mechanisms of action to maximize IOP-lowering efficacy. Overall, iStent technology represents an important component of the current MIGS armamentarium, with its role better clarified as higher-quality and longer-term data becomes available.
